# A Generalized Approach to the Modeling of the Species-Area Relationship

**DOI:** 10.1371/journal.pone.0105132

**Published:** 2014-08-29

**Authors:** Katiane Silva Conceição, Werner Ulrich, Carlos Alberto Ribeiro Diniz, Francisco Aparecido Rodrigues, Marinho Gomes de Andrade

**Affiliations:** 1 Departamento de Matemática Aplicada e Estatística, Instituto de Ciências Matemáticas e de Computação, Universidade de São Paulo, São Carlos, São Paulo, Brazil; 2 Department of Animal Ecology, Nicolaus Copernicus University in Toruń, Toruń, Poland; 3 Department of Statistics, Federal University of São Carlos, São Carlos, Brazil; Universita' del Piemonte Orientale, Italy

## Abstract

This paper proposes a statistical generalized species-area model (GSAM) to represent various patterns of species-area relationship (SAR), which is one of the fundamental patterns in ecology. The approach enables the generalization of many preliminary models, as power-curve model, which is commonly used to mathematically describe the SAR. The GSAM is applied to simulated data set of species diversity in areas of different sizes and a real-world data of insects of *Hymenoptera* order has been modeled. We show that the GSAM enables the identification of the best statistical model and estimates the number of species according to the area.

## Introduction

The variation in the number of species with area, known as species-area relationship (SAR), is one of the most important ecological patterns [1]. The models of SAR enable the prediction of the number of species that coexist and share resources, as well as the impact of the extinction of species caused by habitat loss. Sampled data for a single species, or all species of a specific trophic level within a particular site have shown that the SAR has a well-defined shape, most often described by power and exponential curves [2]. The number of species in an area increases with increasing island area, but the rate of increase slows for larger islands. Many hypotheses have been proposed to explain the SAR [3], [4], [6]. For instance, some are based on the immigration and extinction of species [4], random sampling processes [6] or the Second Law of Thermodynamics [5].

These different hypotheses have generated many mathematical models for the description of the SAR [1]–[3], [7]–[10]. The early models were based on deterministic modeling, which assumes that every set of variable states is uniquely determined by the parameters in the model. For instance, Arrhenius considered that the number of species (*S*) is related to area (*A*) through a power law form [11] (called the power-function), i.e. 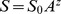 (or 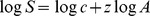), where 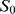 represents the number of species in a unit area (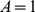) and 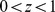. Due to the random nature of the sampled data, statistical modeling is more suitable for SAR description than the deterministic approach [6], therefore, many statistical models have been developed (e.g. [2]). Moreover, statistical models can be thought of as general cases of deterministic models, because the mean value of the random variable of interest yields the results of the deterministic model.

Because there exist many models to address the SAR (e.g. [3], [9], [12]), a natural question is how to select the best model for a given data set. To address this issue, here we integrate different models within a common framework and consider the problem of curve fitting by the transformed generalized linear model (TGLM) [13]. We propose the use of the generalized species-area model (GSAM) to describe the SAR. GSAM includes many models, such as those described in [3], [9] as special cases. We also consider a model that simulates the colonization process of a region by different species and show that the GSMA has best fitted the data in comparison with traditional power-curve models. Finally, we use the data on the cumulative species richness of parasitic *Hymenoptera* from 25 nested plots in a beech forest on limestone [14]. Our results show that the GSAM can determine the best model for the data and estimate the number of species accurately.

## Methods

### Generalized species-area models

In species-area curves, the number of species (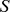) is the dependent variable and the area (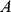) is the explanatory variable. Some mathematical models of SAR propose that the number of species is related to the area as
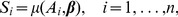
(1)where 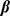 is a *p*-parameter vector [1]. Function 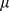 can be derived from laws governing the physical system that gave rise to the data. As such models are deterministic and the properties related to the random nature of variable 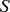 are neglected, the deterministic models are often inadequate due to the stochastic nature of the data [6]. The statistical modeling usually assumes that Eq. 1 can be written as
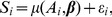
(2)where 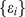 are independent and identically distributed random noises (*i.i.d*) — usually, 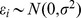. Note that the mode in Eq. 2 is a generalization of the determinic model, i.e. 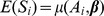. The model given by Eq. 1 can be understood as a particular case of the model 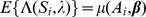, where 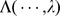 is a monotonic transformation and 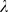 is a scalar parameter defining such a transformation. For instance, in cases whose data suggested by Eq. 2 are unsatisfactory, the experimenter can assume a model with logarithmic transformation, i.e.



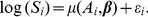
(3)This paper proposes a new model called generalized species-area model (GSAM), which is based on the TGLM approach proposed in [13]. The GSAM works with a general parametric family of transformations from the dependent variable 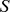 to 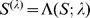 and postulates that the transformed random variable 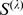 follows a continuous probability distribution belonging to the exponential family. Furthermore, the GSAM assumes that there exists some 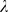 value such that 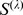 satisfies the usual assumptions of the generalized linear models (GLM) [15].

A suitable choice of the family of transformations enables the representation of power-curves, their recent extensions (see [11], [16]–[19]), the models presented in [2], [3], [9] and the logarithmic model described in [16] as special cases of the GSAM. We have considered the Box-Cox power transformation [20], which is effective at turning skewed unimodal distributions into nearly symmetric normal-like distributions.

Let 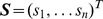 be the vector of observations. By using
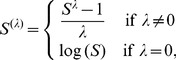
(4)we can obtain the transformed observations 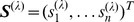. The GSAM assumes that there exists some 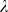 value such that the transformed random variables 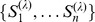 can be considered independently distributed. Each 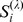 follows an exponential family distribution with a probability density function of the form
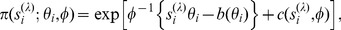
(5)where 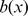 and 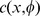 are appropriate known functions. The dispersion parameter 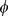 is assumed to be the same for all observations. The mean and variance of 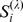 are, respectively, 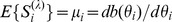 and 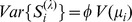, where 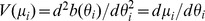 is the variance function. Parameter 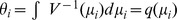 is a known one-to-one function of 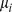.

The GSAM also considers a systematic component given by
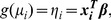
(6)where the link function 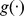 is a known one-to-one continuously differentiable function and 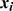 is a specified vector (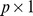) of the explained variables, which include the area, known functions of the area, and other environmental variables. Matrix 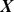 whose rows are vectors 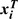, 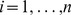, is a specified 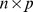 model matrix of full rank 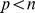 and 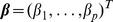 is a set of unknown linear parameters to be estimated. The link function is assumed to be monotonic and differentiable.

The GSAM proposed here considers three components of structural importance: (i) the Box-Cox family of transformations (Eq. 4) in association with a more general form for the distribution of the transformed variable 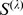 (Eq. 5); (ii) a linear predictor function and (iii) a possible nonlinear link function for the regression parameters (Eq. 6). Moreover, when the variance function 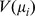 is not constant, i.e. when the variance is correlated with mean 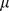, some distributions of the exponential family enable the handling of data presenting heteroscedasticity. In this context, GSAM is a generalization of the previous mathematical models that describe the SAR.

#### Species-area relationship models

Many models have been proposed for the description of the SAR and some can be linearized by a logarithmic transformation of the response variable (i.e. diversity of species). Models that are special cases of the GSAM have the following properties: (i) the transformation parameter in Eq. 4 is 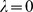, i.e. a log-transformation is adopted, 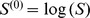 and 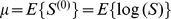 or no transformation is considered for variable 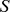, i.e. we assume 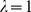 and 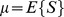; (ii) the distribution in Eq. 5 is the normal distribution; and (iii) the link function is the identity function, 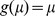 and the systematic component in Eq. 6 is given by 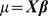. The elements of matrix 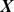 may be area 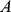, 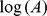 or additional variables, as in [12]. Very simple forms of the systematic component are given by 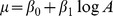 or 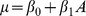. This special case of the GSAM can be understood as the particular cases proposed in [11], [16]. A list of some models of SAR is provided.

1. Considering the stochastic nature of variable 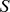, which represents the number of species, the power model proposed by Arrhenius [11] can be written as,

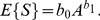
(7)The logarithm of variable 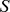 yields
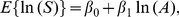
(8)where the parameters of the power-curve in log-log space are 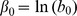 and 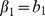.

2. The persistence model (P1-full) proposed by Plotkin et al. [17] is given by
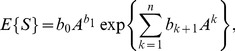
(9)or, considering the logarithm of variable 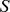,
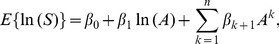
(10)where 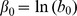, 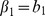 and 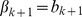, 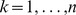. A special case, when 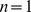 (P1 model [17]) is given by
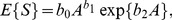
(11)or, considering the logarithm of variable 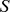,
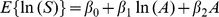
(12)where 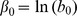, 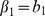 and 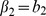.
3. The persistence model proposed by Ulrich and Buszko [18], [19] is given by
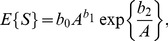
(13)or, considering the logarithm of variable 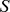,
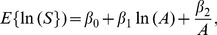
(14)where 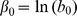, 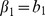 and 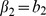.
4. The polynomial power-function model proposed by Chiarucci et al. [21] is defined as
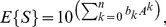
(15)or, considering the logarithm of variable 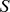,
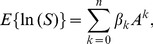
(16)where 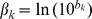, 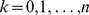. The quadratic power-function model proposed in [21] considers 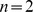, i.e.
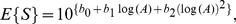
(17)or, considering the logarithm of variable 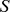,
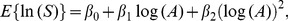
(18)where 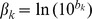, 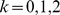.

Some species-area relationships may also be represented by linear functions, specifically:

5. Linear model proposed by MacArthur and Wilson [4]

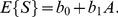
(19)
6. Logarithmic function proposed by Gleason [16]

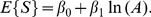
(20)
7. Quadratic logarithmic function proposed by Gitay et al. [22]

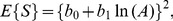
(21)or
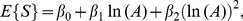
(22)where 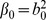, 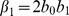 and 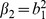

8. General power-logarithmic function proposed by Gitay et al. [22]

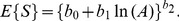
(23)If 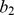 is any real number and 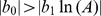, from Newton's generalized binomial expansion we obtain
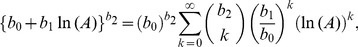
(24)where the binomial coefficients with an arbitrary upper index can be defined as



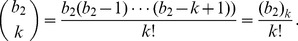
(25)Therefore, the logarithmic function model can be written as
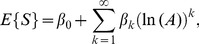
(26)where 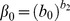, 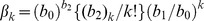.

#### Full-scale generalized species-area relationship model

The right side of all equations presented in the previous section always involves polynomial terms, such as 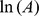, 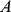 and/or 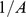. Here, we propose a generalization of these models by considering the right side of the full-scale model consists of three polynomials, i.e.
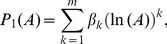
(27)

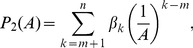
(28)and 
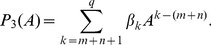
(29)The left sides of those equations have the number of species (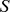) or 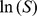. In order to generalize them, we have assumed that 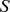 is a random variable and considered the Box-Cox power transformation (Eq. 4).

The curves defined by the GSAM assume a linear predictor function and a nonlinear link function 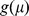 for the systematic component (Eq. 6). The systematic component of the GSAM is given by
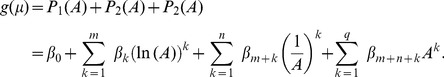
(30)


The GSAM has other models as special cases. For instance, the persistence function, P2-full model, is a special case of GSMA if we consider the identity link function 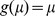, where 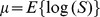, i.e.
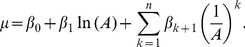
(31)


The persistence model, P2-full, can be written as
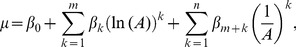
(32)where 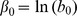, 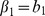 and 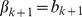, 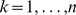.

Although the model defined by Eq. 30 has a large number of parameters (theoretically, it can have an infinite number of parameters), in practice the fitted models have no more than six parameters. The advantage of such a model is that it enables the formulation of hypothesis testing for the choice of the parameters to be removed from those that are significant for better describing the SAR.

#### Model fitting

The parameters to be estimated in the GSAM are 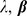 and 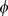 (Eqs. 4 and 6). In order to obtain maximum likelihood estimates for the vector of parameters 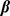 and dispersion parameter 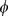, we have defined a profiled likelihood function for 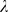 and used the same algorithm proposed in [13]. By assuming the model given by Eq. 5, the log-likelihood function for the vector of the transformed observations 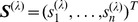 can be written as
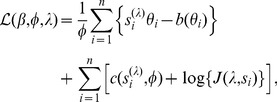
(33)where 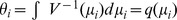 and 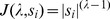, 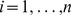 is the Jacobian of the transformation from 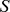 to 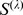.

The procedure described in [13] is used for making inferences about parameters (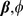) first assuming that 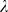 is fixed and obtains the log-likelihood equations for estimating 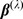 and 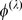. The maximum likelihood estimates (MLE) of 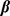, 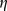, 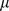 and 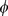 for a given 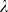 are denoted by 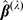, 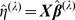, 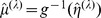 and 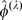, respectively. 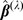 can be calculated, without knowledge on 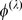, adjusting the GSAM (Eqs. 5–6) to 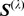 by iteration.

The iteration starts with an initial set of values 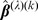, 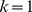, used to evaluate 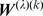 and 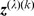, where 
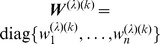
 is a diagonal matrix with weights
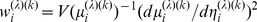
(34)and 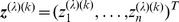 is a working vector whose components are given by



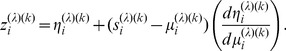
(35)The next estimate 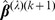 can be obtained by
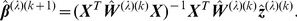
(36)


This new value is used to update 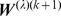 and 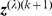 and the procedures are repeated until convergence has been achieved.

Estimating parameter 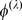 is more difficult than estimating 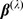. In principle, 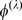 could also be estimated by maximum likelihood, although there may be practical difficulties associated with this task for some members of Eq. 5. Details about the technique used for finding the MLE 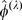 for a fixed 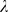 can be found in [13].

In order to obtain the MLE 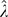, we replace MLE 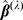 and 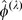 in (33), which results in the profile log-likelihood function 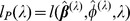. The plot of the profile likelihood function 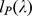 against 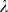 for a sequence of values of 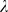 numerically determines the MLE for 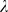. Once the MLE for 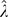 has been obtained, it can be used to produce the unrestricted estimates 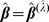 and 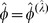.

Assuming that the estimated 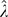 is known, the confidence intervals for parameters 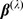 and 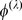 can be calculated in the usual context of the GLM and using the adjusted values 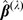 and 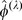. We consider the approximate covariance matrix of 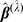 and the variance of 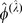 given in [13] to make inferences about these parameters. Here, we have considered the gamma, Gaussian and inverse Gaussian distributions for the probability density function (Eq. 5)

We also performed likelihood ratio (LR) tests [23] using a statistic 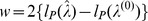, which has an asymptotic 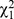 distribution for testing 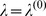 and constructed a large sample confidence interval for 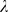 by inverting the LR test.

## Results and Discussion

### Simulation of the colonization process of a region

**Figure 1:**
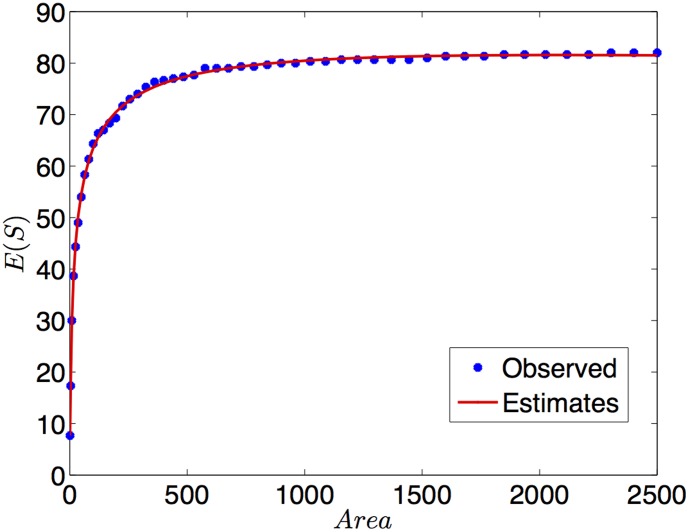
Data and fitted curve obtained by the GSMA model for the simulated species-area relationship.

The parameters of SAR curves are determined from the survey data. As a proof of concept we first used simulated data for 80 species placed in a 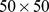 cell lattice according to a neutral model [24] without dispersal limitation, as applied in [25]. This lattice was then resampled so as to establish the shape of the SAR (Figure 1).

The adjusted models are variations of the full-scale model with six parameters, given by
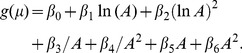
(37)


The following canonical link functions were considered: (i) 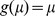 for the normal GSAM, (ii) 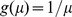 for the gamma GSAM and (iii) 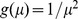 for the inverse Gaussian GSAM. Moreover, the traditional models presented in the previous section were considered by assuming that the random variable 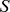 is normally distributed. We could estimate the mean of the transformed data 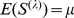, but to predict the expected value of the untransformed dependent variable 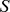, when the GSAM is adjusted to the data, 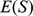 must be estimated. The dependent variable 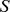 can be explained by subtracting 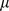 on both sides of Eq. 4 and solving this equation for 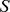. When 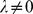 we can write
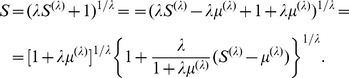
(38)


The expected value of the species number 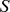, on the original scale, can be evaluated by a first-order approximation of the binomial expansion (Eq. 38), as given in detail in [13]:
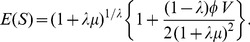
(39)


The best model can be chosen by using the AIC and BIC criteria [26], which are measurements of the relative goodness of fit of a statistical model for a given set of data. The mean square error (MSE) and mean absolute percent error (MAPE) are given, respectively, by
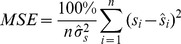
(40)and
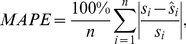
(41)where 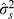 is the sample variance of 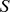 and 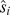 is the estimate of 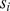 given by Eq. 39.

Table 1 shows some of the traditional models adjusted with normal error. The selected model was the logarithmic quadratic function proposed by [21], with minimum 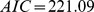, 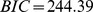, 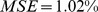 and 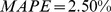.

**Table 1:** Some of the traditional models adjusted with Gaussian errors.

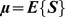	Selection Criteria				
Models	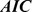	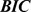	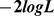	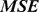	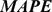
[4]	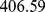	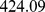	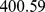	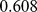	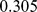
[16]	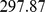	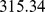	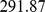	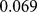	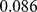
[22]	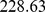	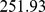	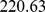	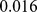	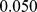

Table 2 shows the selected models adjusted by the GSAM with normal, gamma and inverse Gaussian (I.G.) distributions. Note that the adjusted models are variations of the full-scale model.

**Table 2:** The GSAMs fitted with different models according to the likelihood method.

Models	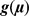	Systematic component
normal	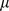	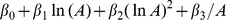
gamma	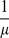	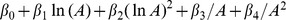
I.G.	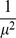	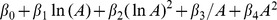

Table 3 shows the GSAM models fitted with 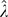 adjusted by the profile likelihood. The selected normal GSAM has minimum 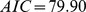 and 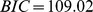, which are the lowest values among the adjusted models. 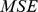 and 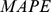 of the model are also smaller than those of the adjusted model with gamma and inverse Gaussian distributions. The adjusted value of parameter 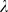 is 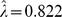 with confidence interval 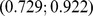. Because 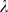 is different from zero or one, there is a significant difference between the results achieved with this model or by using the traditional models given in Table 1. Therefore, for this data set, the normal GSAM is the model that has best fitted the analyzed data. The MLE estimates of the systematic component and standard-deviation (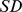) of the systematic component are shown in Table 4.

**Table 3:** Selection criteria for the GSAMs fitted with 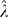 adjusted according to the likelihood method.

Model	Parameter	Selection Criteria				
	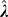	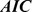	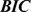	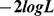	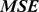	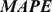
normal	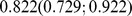	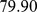	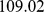	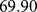	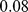	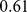
gamma	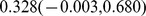	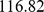	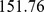	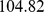	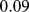	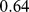
I.G.	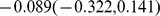	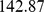	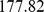	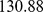	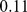	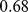

**Table 4:** Normal GSAM model fitted by the systematic component shown in Table 2.

	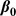	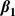	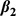	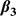
Coefficients	−10.0405	14.1548	−0.9251	15.3104
(SD)	(0.4593)	(0.1713)	(0.0154)	(0.5575)

Figure 1 shows the systematic observation of SAR on the original scale and the fitted curve with the adjusted GSMA models. 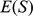 was calculated by Eq. 39 and for the adjusted GSAM we obtained 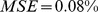 and 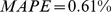, respectively.

### Application to real data

The GSAM model was applied to a data set that consisted of 25 observations of parasitic insects of the *Hymenoptera* order in a beech forest on limestone. *Hymenoptera* is one of the largest orders of insects that comprise sawflies, wasps, bees and ants. The total number of Hymenopteran species in Europe exceeds 20,000. The data considered here contain the summary of a long-term study of the ecology of parasitic Hymenoptera in a German beech forest, i.e. the Göttingen forest, which is approximately 120 years old and has grown over a ground limestone. The climate of the forest is typical of Central Europe and the work area covered approximately four acres. The study was conducted for 8 years (starting in 1980) in 144 square meters of forest soil.

The analysis of the SAR for Hymenoptera is essential, because the insects that belong to this order are the most important environmental agents fundamental for nutrient recycling and control of harmful species. The group is ubiquitous and it is common sense to assume that there is at least one species of parasitic insects for each species of herbivore insects [14]. Many of such species can be considered for the biological control of plague in agriculture. For instance, wasps, from *Symphyta* suborder, are plague conifers in the Northern hemisphere and several species of ants cause losses of millions of dollars for agriculture. Such insects act as special indicators and enable the inference of the diversity of arthropods of a broad spectrum of niches. *Hymenoptera* parasitoids are sensitive to environmental pollution, therefore fluctuations in their population are observed earlier than in their hosts [27]. This sensitivity makes this group an ideal candidate for studies on conservation. Therefore, the knowledge on how the number of species scales with area is fundamental for the prediction of the impact of such insect parasitic on both ecosystems and agriculture.

Table 5 shows the species richness in different sample areas (see also [14]). We modeled the data by taking into account all the models presented in previous sections. The results show that the normal GSAM with 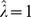 was the best fitted model. No transformation of the original data was necessary:

**Table 5:** Richness of *Hymenoptera* species in different sample areas (*m* ^2^).

Area	Species	Area	Species	Area	Species	Area	Species	Area	Species
1	55	6	133	12	246	20	274	36	379
2	89	6	157	12	204	20	310	49	409
4	167	6	147	12	200	24	311	61	454
4	116	6	146	16	258	24	311	73	473
4	148	8	226	16	260	30	344	89	521



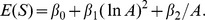



**Figure 2:**
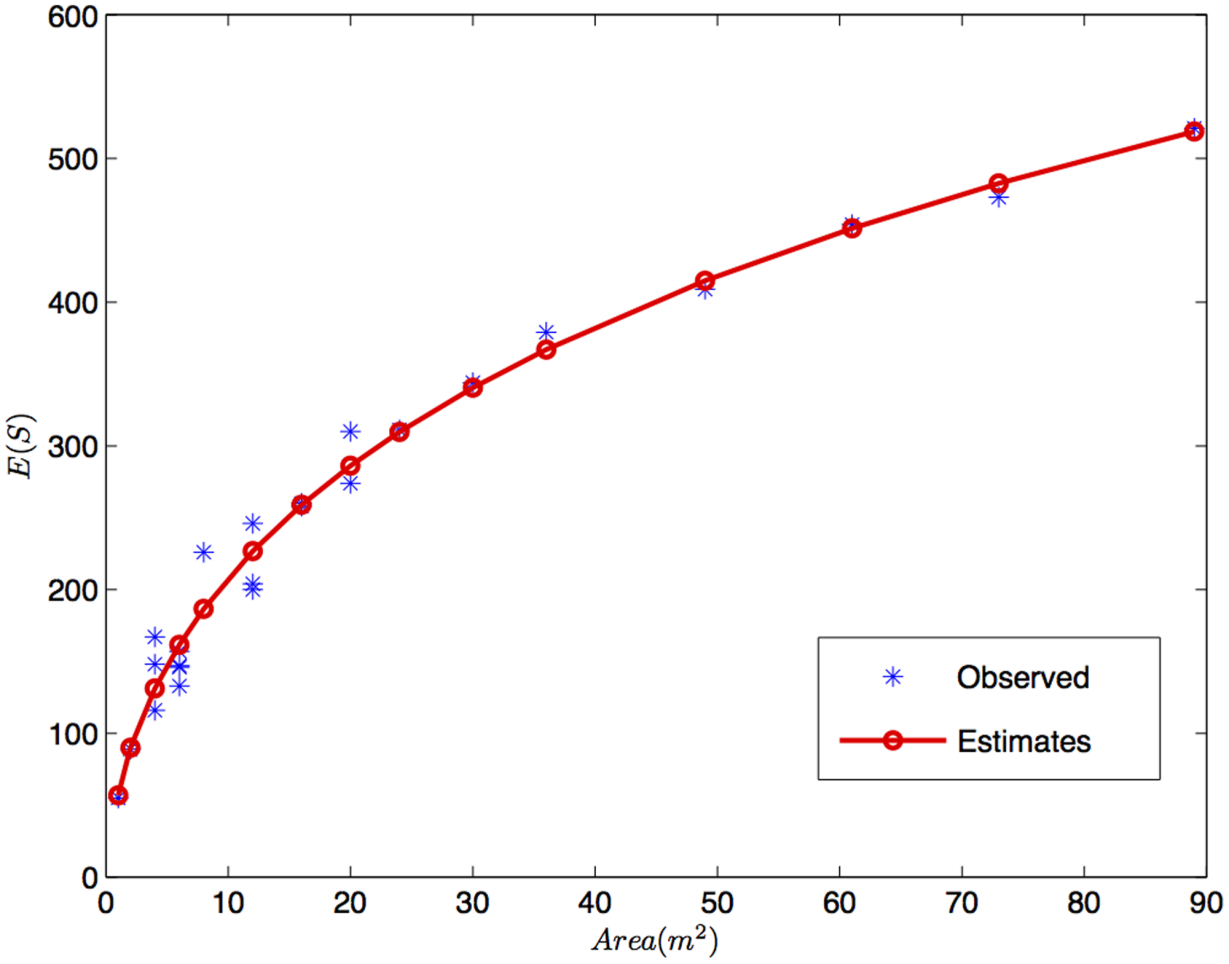
Data and fitted model for the real species-area relationship of *Hymenoptera* in a beech forest on limestone.

The parameters of the fitted model are shown in Table 6. The fitted mean together with the data provided in Table 5 are shown in Figure 2. The adjusted model resulted in 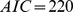, 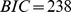, 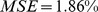 and 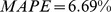, therefore, the GSMA model has proved very accurate. Our fit has provided a very good description of the increase observed in species richness and differs from the simple power-function presented in [14]. Interestingly our best fitting model includes features of the modified persistence model [18], but it has not been predicted by any recent macroecological theory, which calls for a fresh look on the patterns and constraints of spatial species distribution.

**Table 6:** Normal GSAM model fitted to SAR of *Hymenoptera*.

	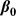	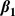	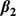
Coefficients	101.9707	20.6625	−46.1669
(SD)	(9.4348)	(0.8211)	(22.3287)

## Conclusions

The generalized species-area model (GSAM) proposed here has provided a generalized model to mathematically describe the SAR. The GSMA can reduce the efforts devoted to finding the best model and can more accurately represent the effect of the area over the diversity of species than the power-curve models commonly used. This fact has been verified in simulated and real-world data.
